# Syndromic disorders caused by gain-of-function variants in *KCNH1*, *KCNK4*, and *KCNN3*—a subgroup of K^+^ channelopathies

**DOI:** 10.1038/s41431-021-00818-9

**Published:** 2021-02-16

**Authors:** Karen W. Gripp, Sarah F. Smithson, Ingrid J. Scurr, Julia Baptista, Anirban Majumdar, Germaine Pierre, Maggie Williams, Lindsay B. Henderson, Ingrid M. Wentzensen, Heather McLaughlin, Lisette Leeuwen, Marleen E. H. Simon, Ellen van Binsbergen, Mary Beth P. Dinulos, Julie D. Kaplan, Anne McRae, Andrea Superti-Furga, Jean-Marc Good, Kerstin Kutsche

**Affiliations:** 1 Division of Medical Genetics, Alfred I. duPont Hospital for Children, Wilmington, DE USA; 2 Department of Clinical Genetics, University Hospitals Bristol and Weston, Bristol, UK; 3 Exeter Genomics Laboratory, Royal Devon & Exeter NHS Foundation Trust, Exeter, UK; 4 College of Medicine and Health, University of Exeter, Exeter, UK; 5 Department of Paediatric Neurology, Bristol Royal Hospital for Children, Bristol, UK; 6 Department of Paediatric Metabolic Medicine, Bristol Royal Hospital for Children, Bristol, UK; 7 Bristol Genetics Laboratory, North Bristol NHS Trust, Bristol, UK; 8 GeneDx, Gaithersburg, MD USA; 9 Invitae, San Francisco, CA USA; 10 Department of Genetics, University of Groningen, University Medical Center Groningen, Groningen, The Netherlands; 11 Department of Genetics, University Medical Center Utrecht, Utrecht, The Netherlands; 12 Section of Genetics and Child Development, Children’s Hospital at Dartmouth, Lebanon, NH USA; 13 Division of Genetics, Birth Defects and Metabolism, Ann & Robert H. Lurie Children’s Hospital of Chicago, Chicago, IL USA; 14 Division of Genetic Medicine, Lausanne University Hospital, Lausanne, Switzerland; 15 Institute of Human Genetics, University Medical Center Hamburg-Eppendorf, Hamburg, Germany

**Keywords:** Paediatric neurological disorders, Genetics research

## Abstract

Decreased or increased activity of potassium channels caused by loss-of-function and gain-of-function (GOF) variants in the corresponding genes, respectively, underlies a broad spectrum of human disorders affecting the central nervous system, heart, kidney, and other organs. While the association of epilepsy and intellectual disability (ID) with variants affecting function in genes encoding potassium channels is well known, GOF missense variants in K^+^ channel encoding genes in individuals with syndromic developmental disorders have only recently been recognized. These syndromic phenotypes include Zimmermann–Laband and Temple–Baraitser syndromes, caused by dominant variants in *KCNH1*, FHEIG syndrome due to dominant variants in *KCNK4*, and the clinical picture associated with dominant variants in *KCNN3*. Here we review the presentation of these individuals, including five newly reported with variants in *KCNH1* and three additional individuals with *KCNN3* variants, all variants likely affecting function. There is notable overlap in the phenotypic findings of these syndromes associated with dominant *KCNN3*, *KCNH1*, and *KCNK4* variants, sharing developmental delay and/or ID, coarse facial features, gingival enlargement, distal digital hypoplasia, and hypertrichosis. We suggest to combine the phenotypes and define a new subgroup of potassium channelopathies caused by increased K^+^ conductance, referred to as syndromic neurodevelopmental K^+^ channelopathies due to dominant variants in *KCNH1*, *KCNK4*, or *KCNN3*.

## Introduction

Potassium (K^+^) channels form a large and diverse group of ion channels, which are encoded by almost 80 genes in the human genome. K^+^ channels show diverse gating properties and have various functions in excitable and non-excitable cells. In neurons, K^+^ channels determine excitability properties and control action potentials in order to maintain excitation homeostasis. K^+^ channels exhibit broad temporal and spatial expression patterns and regulate cellular excitability during development in multiple ways [1–4]. Four α subunits arranged around a central pore segment selective for K^+^ ions are necessary to build a functional potassium channel. According to the number of transmembrane domains, K^+^ channels are divided into three major groups: the “inward rectifier” K^+^ (K_ir_) channel family contains two transmembrane domains, the “two-pore domain leak” K^+^ (K_2P_) channel family has four transmembrane domains, and members of the “voltage-gated” K^+^ (K_v_) family, including “calcium-activated” K^+^ (K_Ca_) channels, have six transmembrane domains. Each α subunit of the channel tetramer has a pore module domain composed of two transmembrane domains, a reentrant pore loop and additional domains which allow responsiveness to different stimuli [4, 5].

Decreased or increased activity of potassium channels caused by loss-of-function and gain-of-function (GOF) variants in the corresponding genes, respectively, underlies a broad spectrum of human disorders affecting the function of the central nervous system, heart, kidney, and other organs [5–8]. While the association of epilepsy and intellectual disability (ID) with variants affecting function in genes encoding potassium channels has been greatly appreciated [2, 5, 6], GOF missense variants in K^+^ channel encoding genes in individuals with syndromic developmental disorders have only recently been recognized [9]. One of these syndromic phenotypes is Zimmermann–Laband syndrome (ZLS) (MIM: 135500), a rare disorder characterized by distinct facial dysmorphism with a bulbous nose and thick ears, gingival enlargement, ID with or without epilepsy, hypo- or aplasia of terminal phalanges and nails, and hypertrichosis [10–13]. Dominant de novo missense variants in *KCNH1* (MIM: 603305), encoding the Eag1 (Kv10.1) channel belonging to the ether-à-go-go family of voltage-gated K^+^ channels, have not only been identified in subjects with ZLS [14], but also in subjects with Temple–Baraitser syndrome (TBS; MIM: 611816) [15]. ZLS and TBS show considerable phenotypic overlap. A total of 23 individuals with *KCNH1* variants affecting function have been reported [14–20]. By studying the biophysical properties of selected KCNH1 mutant channels, a left-shifted current-to-voltage activity and slower deactivation kinetics compared to wild-type channels have been identified, demonstrating a GOF effect for ZLS- and TBS-associated *KCNH1* missense variants [14, 15]. In *KCNK4* (MIM: 605720), which encodes a two-pore domain leak K^+^ channel, the two dominant de novo missense variants p.(Ala172Glu) and p.(Ala244Pro) have been reported in three unrelated subjects. They show a consistent phenotype of characteristic facial dysmorphism, hypertrichosis, epilepsy, developmental delay/ID, and gingival overgrowth for which the acronym FHEIG syndrome has been proposed [21]. KCNK4 (alternative names: TRAAK, K_2P_4.1) belongs to the TRAAK/TREK subfamily of lipid- and mechano-sensitive K_2P_ channels [22]. The two mutant KCNK4 channels showed a higher basal K^+^ conductance and lacked further channel activation in response to mechanical stimuli and arachidonic acid, indicating a GOF effect of the disease-causing amino acid substitutions [21]. The clinical features of the *KCNK4*-related disorder are similar to those observed in individuals with ZLS and TBS [14, 15, 21]. In 2019, we reported de novo GOF missense variants in *KCNN3* (MIM: 602983) [23], encoding the small-conductance Ca^2+^-activated K^+^ channel SK3 that is gated by submicromolar intracellular Ca^2+^ concentrations [24]. KCNN3/SK3 is a homomeric tetramer and part of a multiprotein complex comprising the pore-forming channel subunits, the constitutively bound Ca^2+^ sensor calmodulin (CaM), protein kinase CK2 and protein phosphatase 2A (PP2A) [25]. Electrophysiological recordings comparing KCNN3 wild type and p.(Lys269Glu), p.(Gly350Asp), and p.(Ser436Cys) mutants in a heterologous cell system provided evidence for a GOF effect as an increase in Ca^2+^ sensitivity and a faster activation of the SK3 mutant channels was identified. The three individuals with dominantly acting *KCNN3* variants showed moderate developmental delay or mild-to-moderate ID, coarse facial features, gingival enlargement, hypoplasia of distal phalanges, and aplastic or hypoplastic nails; two individuals had patent ductus arteriosus (PDA). These clinical features overlap with the characteristic ZLS phenotype and with the *KCNK4*-related disorder. For this reason, we propose combining the phenotypes associated with activating *KCNH1*, *KCNK4*, and *KCNN3* alleles to define a new subgroup of potassium channelopathies caused by increased K^+^ conductance [23].

Here, we report eight additional individuals with phenotypes belonging to this K^+^ channelopathy subgroup, including five with *KCNH1* missense variants and three with novel *KCNN3* variants. We review their clinical features and compare them to 22 previously reported individuals with a *KCNH1* GOF allele, the three reported subjects with a dominant *KCNK4* variant, and the  three individuals previously reported with a dominant *KCNN3* variant [14–19, 21, 23]. The aim of this study is to better define and differentiate the syndromic phenotypes associated with variants affecting function in *KCNH1*, *KCNK4*, and *KCNN3* and to delineate the core clinical picture of this subgroup of rare potassium channelopathies.

## Subjects and methods

### Study approval

**Fig. 1:**
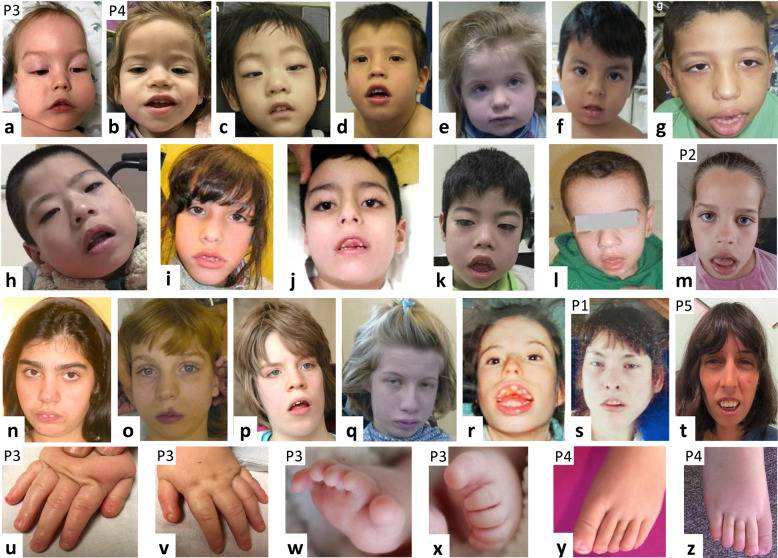
Facial photographs from 20 individuals with a pathogenic *KCNH1* variant. The facial photos are arranged in order of age from youngest to oldest. The five newly reported patients are indicated by P1–P5. Note the hypotonic facial expression, with open mouth posture and inverted V-shape of the upper lip, and apparent ptosis in some individuals. Facial shape elongates with age (third row), but myopathic facial features remain. **a**, **b** Patient 3 (P3; at age 16 months) and patient 4 (P4; at age 1 year 7 months) reported in this study (described in detail in Table 1). **c** Patient at age 3 years reported in [17] (with permission from Springer Nature). **d** Patient at age 4 years reported in [48] (with permission from John Wiley and Sons). **e** Patient at age 4 years 4 months reported in [18] (with permission from Springer Nature). **f** Patient at age 3 years 7 months reported in [49] (with permission from John Wiley and Sons). **g** Patient at age 6 years reported in [17] (with permission from Springer Nature). **h** Patient at age 6 years reported in [17] (with permission from Springer Nature). **i** Patient at age 6 years 10 months reported in [50] (with permission from Wiley and Sons). **j** Patient at age 7 years reported in [14]. **k** Patient at age 8 years reported in [17] (with permission from Springer Nature). **l** Patient at age 9 years reported in [16]. **m** Patient 2 (P2; at age 9 years) reported in this study (described in detail in Table 1) and previously reported in [18] (individual 3). **n** Patient at age 12 years reported in [14]. **o** Patient at age 13 years reported in [18] (with permission from Springer Nature). **p** Patient at age 12 years 8 months reported in [14]. **q** Patient at age 14 years reported in [18] (with permission from Springer Nature). **r** Patient (age unknown) reported in [14]. **s** Patient 1 (P1; at age 14 years) reported in this study (described in detail in Table 1). **t** Patient 5 (P5; at age 34 years) reported in this study (described in detail in Table 1). **u**, **v** Fingers of patient 3 (P3; at age 16 months; as described in detail in Table 1), showing proximally placed hypoplastic thumbs with hypoplastic nails. **w**, **x** Toes of Patient 3 (P3; at age 14 months; as described in detail in Table 1), showing anonychia of toes 1 and 2 and hypoplastic nails on toes 3–5. **y**, **z** Toes of patient 4 (P4; at age 3 years 10 months; as described in detail in Table 1), showing elongated toes with hypoplastic nails.

**Fig. 2:**
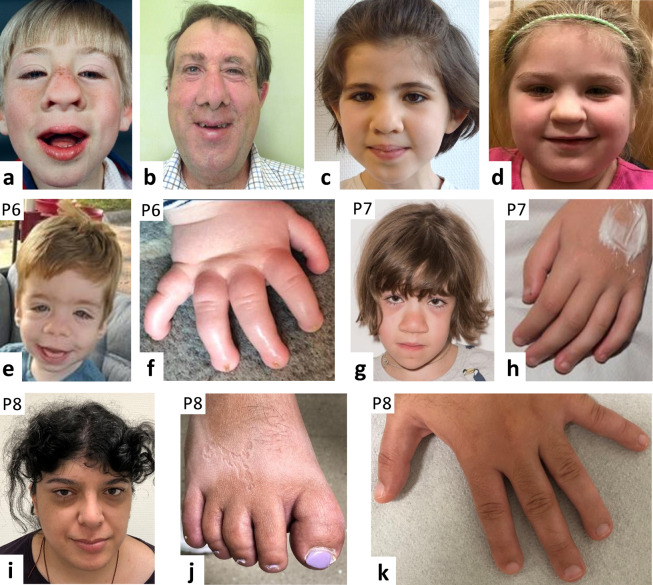
Photographs from six individuals with a pathogenic *KCNN3* variant. The three newly reported patients are indicated by P6–P8. Note the broad nasal tip, wide mouth, and coarse facial features. Same patient as child (**a**; at age 5 years) and as adult (**b**; at age 46 years) after cosmetic facial surgery (previously published in [23]). **c**, **d** Two additional individuals, both aged 5 years, previously reported in [23]. **e** Facial photograph of patient 6 (P6; at age 19 months) reported here (see Table 1 for details) showing epicanthal folds, with distal digital hypoplasia with hypoplastic finger nails (**f**). **g** Facial photograph of patient 7 (P7; at age 9 years) reported here (see Table 1 for details), with hypoplastic finger nails (**h**). **i** Facial photograph of patient 8 (P8; at age 30 years) reported here (see Table 1 for details), showing full lower lip, with hypoplastic toe nails (**j**) and hypoplastic finger nails (**k**).

Informed consent for genetic analyses was obtained from all patients, and genetic studies were performed clinically. The parents of the affected individuals provided informed consent for participation in the study, clinical data and specimen collection, genetic analysis, and publication of relevant findings. Permission to publish and reproduce previously published photographs was provided for all patients shown in Figs. 1 and 2.

### Exome sequencing (ES) and sequence data analysis

ES for patient 2 has been previously published [18]. For patients 1 and 3–8, ES was performed in clinical diagnostic laboratories (for details, see Supplementary information). Parental samples were included in the analysis as available (Table 1).

**Table 1:** Clinical features of patients reported here with a dominant *KCNH1* or *KCNN3* variant.

Patient #	1	2	3	4	5	6	7	8
Gene	*KCNH1*	*KCNH1*	*KCNH1*	*KCNH1*	*KCNH1*	*KCNN3*	*KCNN3*	*KCNN3*
mRNA reference number	NM_172362.2:	NM_172362.2	NM_172362.2	NM_172362.2	NM_172362.2	NM_002249.6	NM_002249.6	NM_002249.6
Variant	c.1487G > A p.(Gly496Glu)	c.1070G > A p.(Arg357Gln)	c.1465C > T p.(Leu489Phe)	c.1060A > G p.(Lys354Glu)	c.1486G > A p.(Gly496Arg)	c.1663G > T p.(Val555Phe)	c.1616_1618del p.(Val539del)	c.859G > T p.(Ala287Ser)
Origin	Parents not tested	de novo	de novo	de novo	Father not tested, not maternal	de novo	de novo	Father not tested, not maternal
Nationality	Caucasian (British)	Caucasian (Dutch)	Caucasian (USA)	Caucasian (USA)	Caucasian (Dutch)	Caucasian (USA)	Caucasian (British)	Swiss-Indian
Sex	F	F	M	F	F	M	F	F
Age^a^	39.2 y	9.75 y	14 m	2.95 y	34 y	1.25 y	11.1 y	30 y
Birth weight (SD)	3300 g (40 wks) (−0.4)	3345 g (40 + 6 wks) (0)	4320 g (39 wks) (+1.9)	3373 g (0)	3870 g (41/42 wks) (+0.8)	2715 g (33 wks) (+2)	3430 g (37 wks) (+1)	ND
Birth length (SD)	ND	52 cm (−0.1)	55.9 cm (+2)	53 cm (+0.8)	53 cm (+0.3)	50 cm (+2)	ND	ND
OFC birth (SD)	35.2 cm (+0.8)	ND	35.5 cm (+1)	ND	ND	36 cm (+3)	35.2 cm (+1.5)	ND
Weight^a^ (SD)	38.0 kg (−2.6)	25 kg (−1)	9 kg (−1.8)	12.5 kg (−0.89)	ND	9 kg (−2)	23.3 kg (−0.4)	89 kg (+1.92)
Height^a^ (SD)	150 cm (−1.7)	122 cm (−2.5)	77.5 cm (0)	94 cm (+0.07)	174 cm (+0.5)	75.6 cm (−1)	124.5 cm (−0.7)	166.5 cm (+0.5)
OFC^a^ (SD)	49.6 cm (−4)	53.5 cm (+1)	46 cm (−0.7)	49.5 cm (+0.32)	55.8 cm (+0.3)	49.8 cm (+2.5)	53.2 cm (+0.9)	58.5 cm (+3.8)
DD/ID	Severe DD; unable to walk independently; severe intellectual disability; no speech	Severe DD/ID; supported walking 21 m; autonomous walking 3.5 y; not able to speak words; able to make sounds	Rolling over at 10 m; not sitting; vocalizing, babbling, no words	Motor and speech delay; skills at 4 m level at age 2.8 y	DD; walking independently around 2 y; speech delay, improvement after ear tubes at 2.5 y; moderate ID	DD with sitting independently at 12 m; no spoken words at 15 m	Mild DD; walked at 2.5 y; first words at 18 m; now good speech and language; but requires support in school	DD; walked at 18 m; first words at 2–3 y; special education; mild-to-moderate ID: speaks in sentences, has basic reading skills
Tonus	Increased tone in limbs with brisk reflexes	Hypotonia; hyperlaxity	Hypotonia	Hypotonia	Hypotonia	Hypotonia	Generalized hypotonia; reflexes difficult to elicit	Mildly increased tone with brisk reflexes
Seizures	Yes, prenatal onset and after birth; initially myoclonic jerks and later a mixed seizure type; improved but not stopped by medication	Yes, onset at 2.75 y; mixed seizure types (focal and diffuse) and absences	No	Yes (age 4 wks)	Yes, onset shortly after birth with generalized tonic-clonic seizures; several times status epilepticus after stopping medication or inadequate doses	No	No	Unclear (see section “Other anomalies”)
EEG	Abnormal	Abnormal	ND	Abnormal	Multifocal abnormalities	ND	Normal	Normal
MRI scan	Microcephaly, mild cerebral atrophy, mild cerebellar hypoplasia (15.2 y)	Somewhat prominent perivascular spaces and high collateral sulcus, further normal	Normal	Normal	ND	Enlarged extra-axial fluid spaces	Normal	Agenesis of corpus callosum
Hearing	Normal	Normal	Passed newborn hearing screen; mild/moderate HL with effusion requiring PE tubes	Normal	Normal	Normal	Normal	Normal
Eye findings	Visual inattention in infancy, gradually improved; left convergent squint	Normal	Normal structural eye exam; severe cortical visual impairment	No evaluation	Normal vision, no evaluation	Normal	Normal	Strabismus
Craniofacial dysmorphism	Narrow face; upslanted palpebral fissures; prominent chin; tented upper lip vermilion; highly arched palate	Hypotonic facies; prominent forehead with bitemporal narrowing; mild eversion of lateral part lower eyelids; protruding maxilla and mandible; wide mouth; full lips; open mouth; tongue protrusion	Hypotonic facies; long face; large jowls; micrognathia and high palate (Pierre–Robin without cleft); arched eyebrows; very long eyelashes; epicanthal folds; appearance of widened inner canthal distance; flat nasal bridge; short upturned nose; tented upper lip	Coarse face; epicanthal folds; slightly broad nasal tip	At adult age: long and narrow face; full hair; hollow cheeks; downslanted palpebral fissures; high nasal bridge; large nose; high-arched palate; open mouth appearance; retrognathia; in childhood more round face with full cheeks	Coarse face; synophrys; low nasal bridge; downslanted palpebral fissures; epicanthal folds; long eyelashes; broad nasal tip	Thick hair and eyelashes; low anterior and posterior hair lines; puffy periorbital region; prominent nose with broad tip; depressed nasal bridge; full cheeks and lips; 3 dimples in left cheek	Coarse face; uplifted earlobes; square-shaped helices; low anterior hairline; long eyelashes, high-arched palate; malpositioned teeth; full lower lip
Gingival enlargement	Yes	Yes	Marked (maxilla)	Yes, at age 2 y	Yes	Marked (surgical reduction)	Yes	No
Skeletal abnormalities of hands and feet	Long and thin hands; proximal placement of thumbs; long narrow feet; long toes with overriding of 2nd toes over 3rd	Hands: slight tapering of fingers; broad thumbs; short distal phalanges of fingers. Feet: broad halluces	Proximally placed and hypoplastic thumbs; tapering of second toe	No signs of skeletal anomaly on hands (radiographs) and feet	Short distal phalanges; hypermobile thumbs; wide forefoot; mild hallux valgus	Distal digital hypoplasia; long great toes	Slight shortening of fingers distally; tapering of 5th fingers; toes unremarkable	Broad big toes; distal phalanges of the toes appear foreshortened
Aplastic or hypoplastic nails	Nails present, but hypoplastic and concave on the fingers	Hands: small nails/slight hypoplasia of nails. Feet: small nails/slight hypoplasia of nails of toes 1 and 5	Anonychia of toes 1 and 2 and hypoplastic nails on toes 3–5; hypoplastic and dysplastic thumb nails	Elongated toes with hypoplastic nails, especially on the 5th toes	Hypoplastic nails	Hands: aplastic nails of thumbs; extreme nail hypoplasia of fingers 2–5. Feet: nail aplasia of all toes	Small nails, which are concave and grow slowly	Hypoplastic nails
Scoliosis	Yes, severe	No	No	Moderate thoracolumbar scoliosis; increased thoracolumbar kyphosis	Mild	ND	No	No
Hypertrichosis	Normal scalp hair and no generalized hypertrichosis	Thick scalp hair; high anterior hairline; long eyelashes	Very long eyelashes	None	Thick scalp hair; hairy legs but no generalized hypertrichosis	Synophrys	Thick scalp hair, but minimal on trunk and extensor surfaces of limbs	Hypertrichosis on face and trunk; low anterior hairline
Liver findings	Liver function normal	Slightly elevated ASAT (84 U/L) and ALAT (98 U/L) at 8.8 y; abdominal ultrasound normal at 3 y	ND	ND	ND	ND	Liver function normal	Liver enzymes normal
Other anomalies	Made some developmental progress until 5 y of age, after which, skills were lost; persistent movement disorder with myoclonic jerks, exacerbated by discomfort or fever	Sialorrhea (required surgery); recurrent ear infections; hip dysplasia; constipation; sleep problems (nitrazepam treatment); behavior problems: can be aggressive and can harm herself by pulling out her hairs; features of autism	Moderate tracheomalacia	None	Bipolar disorder (with a psychotic episode) treated with antipsychotic medication; right bundle branch block; right axis deviation; in childhood ataxia and tremor	Polyhydramnios; hypospadias; microcolon; progressive left ventricular dilatation; mild coronary artery dilation	Soft velvety skin with normal skin creases	Inspiratory stridor in infancy; episodes of recurrent vomiting and lowered vigilance compatible with insular epilepsy (not confirmed); antipsychotic medications for possible hallucinations

*ALAT* alanine aminotransferase, *ASAT* aspartate aminotransferase, *DD* developmental delay, *F* female, *HL* hearing loss, *ID* intellectual disability, *M* male, *m* months, *ND* no data, *OCT* optical coherence tomography, *OFC* occipitofrontal head circumference, *PE* pressure equalizer, *wks* weeks, *y* years.

^a^At last examination.

## Results

### Five individuals with a dominant *KCNH1* variant and three individuals with a dominant *KCNN3* variant

Through an international collaboration, we identified five individuals with a heterozygous *KCNH1* missense variant likely affecting function. Clinical and genetic data of the affected individuals are summarized in Table 1. ES revealed the previously described dominant variants p.(Gly496Glu), p.(Arg357Gln), p.(Leu489Phe), and p.(Gly496Arg) in a 39-year-old female (patient 1), a 9-year-old girl (patient 2), a 14-month-old male (patient 3), and a 34-year-old female (patient 5), respectively. Female patient 4, who was 3 years old at last examination, carried the dominant de novo c.1060A > G/p.(Lys354Glu) variant, which has not been reported previously. None of the variants was identified in the gnomAD database. Adult patient 1 had microcephaly, severe ID, absence of speech, and increased tone in limbs. She had a prenatal onset of seizures that persisted after birth. MRI scan at the age of 15 years revealed mild cerebral atrophy and mild cerebellar hypoplasia. Craniofacial dysmorphism comprised narrow face, upslanted palpebral fissures, tented upper lip vermilion, prominent chin, highly arched palate, and gingival enlargement (Fig. 1). She had long and thin hands with adducted thumbs. Her feet were long and narrow with long toes and overriding of 2nd over 3rd toes. Nails were present but had an abnormal shape. She had severe scoliosis. Patient 2 had developmental delay with independent walking at age 3.5 years. She had mixed seizures and ID with no spoken language at age 9 years. Her hypotonic facial features included bitemporal narrowing, and a wide mouth with full lips (Fig. 1). Facial shape appears to elongate with age in these individuals, as seen in patient 5 who as adult had a very long and narrow face with retrognathia (Fig. 1). Her height and head circumference were within the normal range, but she had short distal phalanges and hypoplastic nails. She had moderate ID and epilepsy.

Patients 3 and 4 showed severe developmental delay (DD) and hypotonia. Patient 4 developed seizures at age 4 weeks, while patient 3 did not have seizures. Both had gingival enlargement. The thumbs of patient 3 were proximally placed and hypoplastic. Craniofacial dysmorphism in patient 3 comprised hypotonic and long face, micrognathia and high palate, arched eyebrows, long eyelashes, epicanthal folds, flat nasal bridge, short upturned nose, and tented upper lip (Fig. 1). Patient 4 showed coarse face with epicanthal folds and slightly broad nasal tip (Fig. 1). Patient 3 had anonychia of 1st and 2nd toes and hypoplastic 3rd–5th toes. His thumb nails were hypoplastic (Fig. 1). Patient 4 had elongated toes with hypoplastic nails (Fig. 1) and thoracolumbar scoliosis and kyphosis.

Three individuals with novel dominant *KCNN3* variants were recruited: the 1-year-old male patient 6 with the de novo c.1663G > T/p.(Val555Phe) missense change, the 11-year-old female patient 7 with the de novo in-frame deletion c.1616_1618del/p.(Val539del), and the 30-year-old female patient 8 with the c.859G > T/p.(Ala287Ser) missense variant (Table 1). None of the variants was identified in the gnomAD database. Patients 6 and 7 showed mild DD and hypotonia, but no seizures. Patient 8 had mild-to-moderate ID, agenesis of the corpus callosum and mildly increased tone with brisk reflexes, seizures were suspected. Patient 6 had coarse face, synophrys, downslanted palpebral fissures, epicanthal folds, very long eyelashes, low nasal bridge, and broad nasal tip (Fig. 2). Craniofacial dysmorphism in patient 7 comprised thick hair with low anterior and posterior hair lines, thick dark eyelashes, full cheeks with three dimples on the left, depressed nasal bridge and prominent nose with broad tip, full lips, and highly arched palate (Fig. 2). Patient 8 had coarse facial features with a low anterior hairline, long eyelashes, a highly arched palate, and a full lower lip (Fig. 2). Gingival enlargement required surgical reduction in patient 6, was present in patient 7 and absent in patient 8. Distal digital hypoplasia and long great toes were observed in patient 6, while patient 7 only had slight distal shortening of fingers. Patient 8 had broad halluces and the distal phalanges of the toes appeared foreshortened with hypoplastic nails. Patient 6 showed anonychia of thumbs and all toes and extreme nail hypoplasia of 2nd–5th fingers. Patient 7 had small and slowly growing nails. Hypospadias and microcolon were observed in patient 6.

### Comparison of clinical features in patients with *KCNH1*, *KCNN3*, and *KCNK4* variants

We collected clinical information for the eight patients with either a *KCNH1* or *KCNN3* variant likely affecting function from this study (Table 1), 22 previously reported individuals with a dominant *KCNH1* variant [14–19],  three previously reported subjects with a dominant *KCNN3* variant [23], and three previously reported subjects with a dominant *KCNK4* missense variant [21]. We did not include the patient with ZLS reported by Guglielmi et al. [20] as the *KCNH1* variant was not described and clinical data were sparse. This brings the total number of studied individuals to 36. As in Bramswig et al. [18] (Table 1) and Fukai et al. [17] (Table 1), we focused on the clinical findings suggestive of ZLS, TBS, and/or FHEIG syndromes, such as neurological, skeletal, and nail abnormalities, as well as gingival enlargement and hypertrichosis (Table 2 and Supplementary Tables 1–3). If facial photographs were available, we evaluated craniofacial dysmorphism in the newly reported and published individuals with a variant affecting function in *KCNH1*, *KCNK4*, or *KCNN3* and defined a facial gestalt associated with dominant variants in either gene (Figs. 1 and 2).

**Table 2:** Frequency of clinical findings in patients with a dominant *KCNH1*, *KCNN3*, or *KCNK4* variant.

Gene	*KCNH1*		*KCNN3*		*KCNK4*	
Total number of patients	27^a^		6^b^		3^c^	
Neurodevelopment						
Mild-moderate DD	3/20	15%	4/4	100%	1/3	33%
Severe DD	18/21	86%	0/4	0%	2/3	66%
Mild-moderate ID	1/23	4%	3/3	100%	1/3	33%
Severe ID	22/23	96%	0/2	0%	2/3	66%
Hypotonia	25/27	96%	4/6	67%	2/3	66%
Seizures/epilepsy	24/27	89%	0/5	0%	2/3	66%
Skeletal abnormalities						
Hypoplastic terminal phalanges of some or all fingers and/or toes	13/17	76%	6/6	100%	ND	ND
Broad thumbs and/or toes	11/24	46%	1/6	17%	ND	ND
Proximal placement and long thumb	14/18	78%	1/6	17%	ND	ND
Long great toes	15/24	63%	2/6	33%	ND	ND
Nails						
Absence or hypoplasia of thumb nail	16/27	59%	5/6	83%	0/3	0%
Absence or hypoplasia of great toe nail	24/27	89%	6/6	100%	0/3	0%
Absence or hypoplasia of other fingers and/or toe nails	16/20	80%	6/6	100%	0/3	0%
Other findings						
Gingival enlargement	15/19	79%	4/6	67%	3/3	100%
Hypertrichosis	3/16	19%	3/6	50%	3/3	100%

*DD* developmental delay, *ID* intellectual disability, *ND* no data.

^a^This study and Simons et al. [15], Kortüm et al. [14], Bramswig et al. [18], Fukai et al. [17], Megarbane et al. [16], Mastrangelo et al. [19].

^b^This study and Bauer et al. [23].

^c^Bauer et al. [21].

#### Neurological features

All 36 individuals had DD or ID. Eighteen of 21 (86%) patients with dominant *KCNH1* variant had severe DD and the level of ID, determined in 23 individuals, was severe in 22 (96%) and mild to moderate in 1 (4%). In the three individuals with dominant *KCNK4* variant, two had severe and one mild-moderate DD and ID. Four subjects with dominant *KCNN3* variant had mild or moderate DD and the three oldest individuals, aged 11, 30, and 46 years, had mild ID. Hypotonia was present in 26/27 (96%) individuals with dominant *KCNH1* variant, in 4/6 (67%) with dominant *KCNN3* variant, and in 2/3 (66%) with dominant *KCNK4* variant. The majority of individuals with dominant *KCNH1* variant had seizures (24/27; 89%), while only two of the three (66%) with dominant *KCNK4* variant developed seizures. Five individuals with dominant *KCNN3* variant did not show epilepsy; in one patient seizures were suspected.

#### Skeletal abnormalities

Finger and toe abnormalities were not reported in the three individuals with dominant *KCNK4* variant [21]. All six individuals with dominant *KCNN3* variant and 13 of 17 (76%) with dominant *KCNH1* variant had hypoplastic terminal phalanges of some or all fingers and/or toes. Broad thumbs and/or toes were present in 11/24 (46%) individuals with dominant *KCNH1* variant, while only 1/6 (17%) with dominant *KCNN3* variant had these limb abnormalities. Proximal placement and long thumb was observed in 14/18 (78%) cases with dominant *KCNH1* and in 1/6 (17%) individuals with dominant *KCNN3* variant. Overall, 15/24 (63%) individuals with dominant *KCNH1* variant and 2/6 (33%) with dominant *KCNN3* variant had long great toes.

#### Nail anomalies

Absent or hypoplastic thumb nail(s) were observed in 16/27 (59%) individuals with dominant *KCNH1* and 5/6 (83%) with dominant *KCNN3* variant. In all individuals (100%) with a dominant *KCNN3* variant, absence or hypoplasia of great toe nail and of other finger and/or toe nails was present. 24/27 (89%) and 16/20 (80%) patients with dominant *KCNH1* variant had absent or hypoplastic great toe nail and anonychia or nail hypoplasia of other fingers and/or toes, respectively. No individual with dominant *KCNK4* variant had nail anomaly [21].

#### Other findings

Gingival enlargement was documented in 15/19 (79%) individuals with dominant *KCNH1*, in 4/6 (67%) individuals with dominant *KCNN3*, and in all three (100%) with dominant *KCNK4* variant. Similarly, all individuals with dominant *KCNK4* variant (100%) had hypertrichosis, while only 3/16 (19%) and 3/6 (50%) with dominant *KCNH1* and *KCNN3* variant, respectively, showed hypertrichosis.

#### Craniofacial dysmorphism

We evaluated facial photographs of 20 here reported and previously published individuals with dominant *KCNH1* variant and identified the following craniofacial features as most common findings: myopathic and long facies, epicanthal folds, broad nasal tip, and open and wide mouth with tented upper lip vermilion (Fig. 1). The three previously reported and three additional individuals with dominant *KCNN3* variant reported here have coarse facial features with thick eyebrows and mild-to-moderate synophrys, prominent nose with a broad nasal tip and triangular nostrils (Fig. 2). Shared craniofacial features in the three individuals with dominant *KCNK4* variant comprise bushy and straight eyebrows, long eyelashes, short philtrum, prominent vermillion, and micrognathia [21].

## Discussion

We studied a total of 36 individuals with a variant likely affecting function in a potassium channel encoding gene, including eight newly reported individuals, and determined the frequency of overlapping clinical features typical for TBS, ZLS, and FHEIG syndromes in the 27 individuals with dominant *KCNH1* variants, six with dominant *KCNN3*, and three with dominant *KCNK4* variants. The data show that the 36 individuals have an overarching clinical picture, however, the phenotypes related to each of the three genes exhibit a distinctive constellation of clinical features that may be recognizable by clinical geneticists. All 36 individuals had DD and/or ID, but of variable degree. While the vast majority of subjects with *KCNH1* variant likely affecting function had severe DD and/or severe ID, all patients with *KCNN3* variant had a milder form of DD and/or ID. The two individuals with the same *KCNK4* variant affecting function [p.(Ala172Glu)] showed severe ID, and they were reported to have nystagmus with bilateral optic hypoplasia. Seizures and/or epilepsy is a typical hallmark of the *KCNH1* disorder (89%). None of the six individuals with dominant *KCNN3* variant had epilepsy, although seizures could not be excluded in one patient. We evaluated skeletal abnormalities and found finger and toe abnormalities in patients with *KCNH1* and *KCNN3* variants likely affecting function. Hypoplasia of terminal phalanges is a typical feature in individuals with dominant *KCNH1* (76%) and *KCNN3* variants (100%). Proximal placement of and long thumb and long great toes were seen in individuals with both dominant *KCNH1* (78% and 63%, respectively) and *KCNN3* variants (17% and 33%, respectively). Broad thumb and/or toe was observed in 46% of individuals with dominant *KCNH1* variant and in 17% with dominant *KCNN3* variant. Nail anomalies, such as absence or hypoplasia of finger and/or toe nails, were present in the majority of individuals with dominant *KCNH1* (59–89%) and in all with *KCNN3* variant. In patients with dominant *KCNK4* variant, nail dysplasia was absent, and data on specific finger and toe abnormalities were not reported. The frequency of gingival enlargement and hypertrichosis that are characteristic clinical features of ZLS was determined. Both features were consistently present in individuals with *KCNK4* variant affecting function and variably present in patients with dominant *KCNN3* (30% for hypertrichosis and 67% for gingival enlargement) or *KCNH1* variant (19% for hypertrichosis and 79% for gingival enlargement). Based on the data, the overarching phenotype associated with dominant *KCNH1*, *KCNN3*, and *KCNK4* variants comprised DD and/or ID, hypotonia, coarsening facial features, gingival enlargement, and hypertrichosis. The greatest clinical overlap was observed between *KCNH1*- and *KCNN3*-related disorders as both are additionally characterized by nail and terminal phalangeal aplasia/hypoplasia and additional thumb and toe abnormalities. The degree of DD and/or ID, presence or absence of seizures, broad thumb or toe, and the facial gestalt may help distinguishing between individuals harboring a *KCNH1* or *KCNN3* variant likely affecting function. On the other hand, the three reported individuals with dominant *KCNK4* variant show distinctive facial features with bushy eyebrows, long eyelashes, short philtrum, and prominent vermillion together with consistent gingival enlargement and generalized hypertrichosis that may prompt clinicians to consider FHEIG syndrome.

We propose to define a subgroup of rare potassium channelopathies, which comprises TBS, ZLS, and FHEIG syndromes, all rare developmental and clinically recognizable disorders caused by GOF variants in genes coding for three different membrane-bound potassium channels. Similarly, Hamilton and Suri [9] suggested the term “electrifying dysmorphology” for a group of dysmorphic syndromes characterized by ID, coarse face, gingival overgrowth, hypertrichosis, and digital/toe anomalies that arise from variants in potassium channel encoding genes. Besides ZLS, TBS, and FHEIG syndromes, Hamilton and Suri [9] discussed Birk–Barel syndrome, Andersen–Tawil syndrome, Keppen–Lubinsky syndrome, and Cantú syndrome (CS). We already noticed clinical overlap of ZLS and CS and found early  DD, hypertrichosis, gingival enlargement, joint laxity, and hypoplasia of terminal phalanges and nails in one or several of the nine recently reported individuals with a dominant variant in *ABCC9* [26]. Dominant variants in *ABCC9* and, rarely, in *KCNJ8*, encoding the regulatory (SUR2) and pore-forming (Kir6.1) subunits, respectively, of ATP-sensitive potassium (K_ATP_) channels cause CS [27–30]. Distinctive craniofacial features of CS, including coarse facial features, low anterior hairline, wide nasal bridge, epicanthal folds, full lips, and hypertrichosis of the forehead [31], can also be seen in individuals with dominant *KCNN3* or *KCNK4* variant (Figs. 1 and 2). Typical CS-associated cardiovascular anomalies, including PDA, mild ventricular hypertrophy, hypertrophy of the ventricular septum, and aorta dilatation, may help distinguishing CS from ZLS and/or FHEIG syndrome [26]. Interestingly, PDA, the most frequent cardiac finding in CS (58%) [31], has also been reported in two of six individuals with dominant *KCNN3* variant, further showing clinical overlap of CS and the *KCNN3*-related phenotype.

The biological processes by which pathogenic variants in genes coding for K^+^ channels lead to complex developmental phenotypes are poorly understood. The *KCNH1* encoded Eag1/K_V_10.1 channel plays a role in cell cycle control and proliferation through cilia disassembly prior to mitosis. Eag1/Kv10.1 hyperactivity due to activating variants has been speculated to cause skeletal and nail malformations through altering signaling pathways involved in morphogenesis, such as the sonic hedgehog pathway [32, 33]. We recently put forward a different hypothesis for the development of digital abnormalities in individuals with *KCNN3* variants affecting function [23]. KCNN3 together with KCNN4 channels function in endothelial Ca^2+^ dynamics to induce vascular tone and blood pressure changes [34–37]. Local activity of KCNN3 in subspaces of endothelial cells modulates vascular tone and blood pressure [36, 38]. Based on these data we speculated that excessive sustained K^+^ conductance caused by increased Ca^2+^ sensitivity of KCNN3 mutant channels leads to enhanced arterial vasodilation and increasing intracapillary pressure during specific stages of embryonic development. This can cause vasodilatory edema, vascular ruptures and/or tissue damage leading to digital hypoplasia or aplasia in individuals with GOF *KCNN3* variants. Further evidence for a role of vascular dilation in the development of nail and phalangeal hypoplasia comes from the malformations seen in children with the fetal-hydantoin syndrome [39]. The syndrome is caused by in utero exposure to hydantoin and/or its derivatives such as phenytoin and nifedipine which belong to antiepileptic drugs. These vasodilating drugs cause distal digital defects in rabbits, when given on day 16 of pregnancy that were preceded by edema, hemorrhage and vascular disruption [40, 41]. In neonates with fetal-hydantoin syndrome, hypoplastic finger and toe nails, digitalized great toe and congenital heart diseases, including pulmonary or aortic valvular stenosis, coarctation of aorta and PDA, are characteristic abnormalities [42–44]. These clinical features show considerable overlap with CS and ZLS. In addition, administration of the K_ATP_ agonist minoxidil causes hypertrichosis as a side effect [45], supporting a link between overactivity of these and other K^+^ channels and the development of hypertrichosis [46, 47]. Similarly, hypertrichosis and gingival hyperplasia are well recognized side effects of phenytoin use (https://www.aesnet.org/sites/default/files/file). Taken together, studies from teratogens and basic research on potassium channel function suggest that overactivity of these ion channels and increased K^+^ conductance underlie the clinical similarities seen in a subgroup of potassium channelopathies characterized by DD/ID, epilepsy, coarse facial features, gingival enlargement, hypertrichosis, and/or nail and phalangeal aplasia or hypoplasia.

## Supplementary information

Supplementary Information
