# Supplementary material for: Syndromic disorders caused by gain-of-function variants in KCNH1, KCNK4, and KCNN3—a subgroup of K+ channelopathies
Source: Eur J Hum Genet. 2021 Feb 16;29(9):1384–95. doi: 10.1038/s41431-021-00818-9 (PMC8440610; doi:10.1038/s41431-021-00818-9)
Supplement: Supplementary file 1 — Supplementary Information [file 41431_2021_818_MOESM1_ESM.docx]

**Supplementary Information**

**Material and Methods**

**Exome sequencing and sequence data analysis**

**Patient 1**

***KCNH1* c.1487G>A/p.(Gly496Glu)**

Trio whole-exome sequencing was performed by the Deciphering Developmental Disorders (DDD) project [1]. Sanger sequencing confirmation of the NM_172362.2(*KCNH1*):c.1487G>A p.(Gly496Glu) variant was carried out in the proband (parents’ DNA samples were not available locally). Variant interpretation and classification was carried out according to the American College of Genetics and Genomics (ACMG) variant classification guidelines [2].

**Patient 3**

***KCNH1*: c.1465C>T/p.(Leu489Phe)**

Genomic DNA obtained from the submitted sample was enriched for coding exons and adjacent splice junctions, generally exons +/- 10 base pairs, using a hybridization-based protocol and sequenced using Illumina technology. These regions were sequenced to an average of ≥50x depth

with minimum call depth of ≥20x. Reads were aligned to a reference sequence (GRCh37) and variants were identified using a bioinformatics pipeline developed and validated at Invitae. Identified variants are filtered and ranked using a proprietary algorithm, which considers known gene-phenotype associations, molecular variant characteristics, zygosity, and population frequency, in the context of the patient's reported clinical presentation. This process is supported by an expertly curated gene-phenotype knowledgebase, as previously described [3]. Variants that may explain some or all of the patient’s provided clinical indication are reviewed, interpreted, and reported by scientists, genetic counselors, and board-certified geneticists according to the guidelines established by the ACMG [2].

**Patients 4 and 6**

***KCNH1*: c.1060A>G/p.(Lys354Glu) and *KCNN3*: c.1663G>T/p.(Val555Phe)**

Using genomic DNA from the proband and parents, the exonic regions and flanking splice junctions of the genome were captured using the IDT xGen Exome Research Panel v1.0. Massively parallel (NextGen) sequencing was performed using an Illumina system with 100bp or greater paired-end reads. Reads were aligned to human genome build GRCh37/UCSC hg19, and analyzed for sequence variants using a custom-developed analysis tool. Additional sequencing technology and variant interpretation protocol has been previously described [4]. The general assertion criteria for variant classification are publicly available on the GeneDx ClinVar submission page.

**Patient 5**

***KCNH1*: c.1486G>A/p.(Gly496Arg)**

DNA samples from the patient and her mother were extracted from peripheral blood. Exomes were enriched using the SureSelectXT Clinical Research Exome V2 (Agilent, elid S30409818, genome build GRCh37) and sequenced on an Illumina Novaseq 6000. The sequencing data was processed with an inhouse developed pipeline, IAP v2.7.1, based on the Genome Analysis Toolkit (GATK v3.8-1-0-gf15c1c3ef) best practices guidelines (<https://zenodo.org/record/2632559#.X225AxKxVaQ>) [5]. The read pairs were mapped with BWA-MEM v0.7.5a (https://arxiv.org/abs/1303.3997), marking duplicates and merging lanes using Sambamba v0.6.5 [6] and realigning indels using GATK IndelRealigner. GATK Haplotypecaller was used to call single nucleotide polymorphisms and indels, creating variant call formatted files. Detected variants were annotated, filtered and prioritized using the Alissa NGS Lab platform (Agilent). Only variants with a minor allele frequency of <0.5% were included in the analysis. The *KCNH1* variant was reported since it was earlier reported in literature in a patient with an overlapping clinical phenotype [7] and its absence in the healthy mother.

**Patient 7**

***KCNN3*: c.1616_1618del/p.(Val539del)**

Whole-exome sequencing (WES) was performed on genomic DNA extracted from leukocytes of the proband and her healthy parents by the Exeter Genomics Laboratory (Exeter, UK). DNA samples were subjected to NGS library preparation with target enrichment with the SureSelect Human All Exon V6 Kit (Agilent). Each captured library was then loaded and sequenced on the NextSeq 500 platform (Illumina). Reads were aligned to human genome build GRCh37/UCSC hg19, and analyzed for sequence variants using a custom-developed analysis tool as previously described [8]. Variants were interpreted and classified according to the ACMG variant classification guidelines [2]. The heterozygous *KCNN3* c.1616_1618del/p.(Val539del) (NM_002249.5) *in-frame* deletion was *de novo* as supported by its absence in 145 and 90 maternal and paternal DNA reads, respectively.

**Patient 8**

***KCNN3:* c.859G>T/p.(Ala287Ser)**

Whole‐Exome Sequencing (WES) was carried out using genomic DNA extracted from leukocytes, on Illumina HiSeq2500 sequencer, with Agilent Sure Select XT Human All Exon V7 capture kit, after appropriate informed consent. Data processing, variants annotation and filtering were made as already described [9]. We analyzed virtual gene panels (neurotransmission, 27 genes; malformation of corpus callosum, 384 genes; mitochondrial diseases, 359 genes; developmental disorders, 1821 genes) and variants were interpreted according to the American College of Genetics and Genomics (ACMG) variant classification guidelines [2]. Confirmation of the variant in the *KCNN3* gene was performed in the proband and her mother by Sanger sequencing.

**Suppl. Table 1. Clinical features of 27 patients with a dominant *KCNH1* variant**

|  | **This study** | **Simons  et al. 2015**  **[10]** | **Kortüm  et al. 2015**  **[7]** | **Bramswig et al. 2016**  **[11]** | **Fukai  et al. 2016**  **[12]** | **Megarbane et al. 2016**  **[13]** | **Mastran-gelo et al. 2016 [14]** | **Total** | **%** |
| --- | --- | --- | --- | --- | --- | --- | --- | --- | --- |
| Patient number | 5 | 6 | 6 | 4 | 4 | 1 | 1 | 27 | 100% |
| **Neurodevelopment** | | | | | | | | | |
| Mild-moderate developmental delay | 3/5 | ND |  |  |  |  |  | 3/20 | 15% |
| Severe developmental delay | 2/5 | ND | 6/6 | 4/4 | 4/4 | 1/1 | 1/1 | 18/21 | 86% |
| Mild-moderate intellectual disability | 1/3 |  |  |  |  |  |  | 1/23 | 4% |
| Severe intellectual disability | 2/3 | 5/5 | 6/6 | 4/4 | 4/4 | 1/1 |  | 22/23 | 96% |
| Hypotonia | 4/5 | 6/6 | 6/6 | 4/4 | 4/4 | 1/1 | 1/1 | 26/27 | 96% |
| Seizures/epilepsy | 4/5 | 6/6 | 6/6 | 3/4 | 4/4 | 1/1 | 0/1 | 24/27 | 89% |
| **Skeletal abnormalities** | | | | | | | | | |
| Hypoplastic terminal phalanges of some or all fingers and/or toes | 3/5 | 5/6 | 4/5 | ND | ND | 1/1 | ND | 13/17 | 76% |
| Broad thumbs and/or toes | 1/5 | 5/6 | 0/4 | 1/4 | 3/4 | 1/1 | ND | 11/24 | 46% |
| Proximal placement and long thumb | 2/5 | 6/6 | 3/4 | 2/2 | ND | 1/1 | ND | 14/18 | 78% |
| Long great toes | 1/5 | 6/6 | 1/4 | 3/4 | 3/4 | 1/1 | ND | 15/24 | 63% |
| **Nails** | | | | | | | | | |
| Absence or hypoplasia of thumb nail | 2/5 | 6/6 | 5/6 | 0/4 | 1/4 | 1/1 | 1/1 | 16/27 | 59% |
| Absence or hypoplasia of great toe nail | 4/5 | 6/6 | 5/6 | 4/4 | 3/4 | 1/1 | 1/1 | 24/27 | 89% |
| Absence or hypoplasia of other fingers and/or toe nails | 5/5 | ND | 5/6 | 2/4 | 2/2 | 1/1 | 1/1 | 16/20 | 80% |
| **Other findings** | | | | | | | | | |
| Gingival enlargement | 5/5 | ND | 5/6 | 4/4 | 0/3 | 1/1 | ND | 15/19 | 79% |
| Hyper­trichosis | 0/5 | ND | 3/6 | ND | 0/4 | 0/1 | ND | 3/16 | 19% |

ND, no data.

**Suppl. Table 2. Clinical features of six patients with a dominant *KCNN3* variant**

|  | **This study** | **Bauer  et al. 2019**  **[15]** | **Total** | **%** |
| --- | --- | --- | --- | --- |
| Patient number | 3 | 3 | 6 | 100% |
| **Neurodevelopment** | | | | |
| Mild-moderate developmental delay | 2/2 | 2/2 | 4/4 | 100% |
| Mild-moderate intellectual disability | 2/2 | 1/1 | 3/3 | 100% |
| Hypotonia | 2/3 | 2/3 | 4/6 | 67% |
| Seizures/epilepsy | 0/2 | 0/3 | 0/5 | 0% |
| **Skeletal abnormalities** | | | | |
| Hypoplastic terminal phalanges of some or all fingers and/or toes | 3/3 | 3/3 | 6/6 | 100% |
| Broad thumbs and/or toes | 1/3 | 0/3 | 1/6 | 17% |
| Proximal placement and long thumb | 0/3 | 1/3 | 1/6 | 17% |
| Long great toes | 1/3 | 1/3 | 2/6 | 33% |
| **Nails** | | | | |
| Absence or hypoplasia of thumb nail | 3/3 | 2/3 | 5/6 | 83% |
| Absence or hypoplasia of great toe nail | 3/3 | 3/3 | 6/6 | 100% |
| Absence or hypoplasia of other fingers and/or toe nails | 3/3 | 3/3 | 6/6 | 100% |
| **Other findings** | | | | |
| Gingival enlargement | 2/3 | 2/3 | 4/6 | 67% |
| Hypertrichosis | 1/3 | 2/3 | 3/6 | 50% |

**Suppl. Table 3. Clinical features of three patients with a dominant *KCNK4* variant**

|  | **Bauer  et al. 2018**  **[16]** | **%** |  |
| --- | --- | --- | --- |
| Patient number | 3 | 100% |  |
| **Neurodevelopment** | | |  |
| Mild-moderate DD | 1/3 | 33% |  |
| Severe DD | 2/3 | 66% |  |
| Mild-moderate ID | 1/3 | 33% |  |
| Severe ID | 2/3 | 66% |  |
| Hypotonia | 2/3 | 66% |  |
| Seizures/epilepsy | 2/3 | 66% |  |
| **Skeletal abnormalities** | | | |
| Hypoplastic terminal phalanges of some or all fingers and/or toes | ND | ND |  |
| Broad thumbs and/or toes | ND | ND |  |
| Proximal placement and long thumb | ND | ND |  |
| Long great toes | ND | ND |  |
| **Nails** | | | |
| Absence or hypoplasia of thumb nail | 0/3 | 0% |  |
| Absence or hypoplasia of great toe nail | 0/3 | 0% |  |
| Absence or hypoplasia of other fingers and/or toe nails | 0/3 | 0% |  |
| **Other findings** | | |  |
| Gingival enlargement | 3/3 | 100% |  |
| Hyper­trichosis | 3/3 | 100% |  |

ND, no data.

**References:**

1. Deciphering Developmental Disorders S. Large-scale discovery of novel genetic causes of developmental disorders. Nature. 2015;519:223-228.

2. Richards S, Aziz N, Bale S, Bick D, Das S, Gastier-Foster J, et al. Standards and guidelines for the interpretation of sequence variants: a joint consensus recommendation of the American College of Medical Genetics and Genomics and the Association for Molecular Pathology. Genet Med. 2015;17:405-424.

3. Clark MM, Hildreth A, Batalov S, Ding Y, Chowdhury S, Watkins K, et al. Diagnosis of genetic diseases in seriously ill children by rapid whole-genome sequencing and automated phenotyping and interpretation. Sci Transl Med. 2019;11.

4. Retterer K, Juusola J, Cho MT, Vitazka P, Millan F, Gibellini F, et al. Clinical application of whole-exome sequencing across clinical indications. Genet Med. 2016;18:696-704.

5. McKenna A, Hanna M, Banks E, Sivachenko A, Cibulskis K, Kernytsky A, et al. The Genome Analysis Toolkit: a MapReduce framework for analyzing next-generation DNA sequencing data. Genome Res. 2010;20:1297-1303.

6. Tarasov A, Vilella AJ, Cuppen E, Nijman IJ, Prins P. Sambamba: fast processing of NGS alignment formats. Bioinformatics. 2015;31:2032-2034.

7. Kortüm F, Caputo V, Bauer CK, Stella L, Ciolfi A, Alawi M, et al. Mutations in KCNH1 and ATP6V1B2 cause Zimmermann-Laband syndrome. Nat Genet. 2015;47:661-667.

8. Le Fevre A, Baptista J, Ellard S, Overton T, Oliver A, Gradhand E, et al. Compound heterozygous Pkd1l1 variants in a family with two fetuses affected by heterotaxy and complex Chd. Eur J Med Genet. 2020;63:103657.

9. Royer-Bertrand B, Castillo-Taucher S, Moreno-Salinas R, Cho TJ, Chae JH, Choi M, et al. Mutations in the heat-shock protein A9 (HSPA9) gene cause the EVEN-PLUS syndrome of congenital malformations and skeletal dysplasia. Sci Rep. 2015;5:17154.

10. Simons C, Rash LD, Crawford J, Ma L, Cristofori-Armstrong B, Miller D, et al. Mutations in the voltage-gated potassium channel gene KCNH1 cause Temple-Baraitser syndrome and epilepsy. Nat Genet. 2015;47:73-77.

11. Bramswig NC, Ockeloen CW, Czeschik JC, van Essen AJ, Pfundt R, Smeitink J, et al. 'Splitting versus lumping': Temple-Baraitser and Zimmermann-Laband Syndromes. Hum Genet. 2015;134:1089-1097.

12. Fukai R, Saitsu H, Tsurusaki Y, Sakai Y, Haginoya K, Takahashi K, et al. De novo KCNH1 mutations in four patients with syndromic developmental delay, hypotonia and seizures. J Hum Genet. 2016;61:381-387.

13. Megarbane A, Al-Ali R, Choucair N, Lek M, Wang E, Ladjimi M, et al. Temple-Baraitser Syndrome and Zimmermann-Laband Syndrome: one clinical entity? BMC Med Genet. 2016;17:42.

14. Mastrangelo M, Scheffer IE, Bramswig NC, Nair LD, Myers CT, Dentici ML, et al. Epilepsy in KCNH1-related syndromes. Epileptic Disord. 2016;18:123-136.

15. Bauer CK, Schneeberger PE, Kortüm F, Altmuller J, Santos-Simarro F, Baker L, et al. Gain-of-Function Mutations in KCNN3 Encoding the Small-Conductance Ca(2+)-Activated K(+) Channel SK3 Cause Zimmermann-Laband Syndrome. Am J Hum Genet. 2019;104:1139-1157.

16. Bauer CK, Calligari P, Radio FC, Caputo V, Dentici ML, Falah N, et al. Mutations in KCNK4 that Affect Gating Cause a Recognizable Neurodevelopmental Syndrome. Am J Hum Genet. 2018;103:621-630.
